# Development of an internet-based intervention to improve health professionals’ counseling skills around adolescent weight management in Indonesia

**DOI:** 10.1371/journal.pone.0294986

**Published:** 2023-12-07

**Authors:** Fransisca Handy Agung, Rini Sekartini, Nani Sudarsono, Aryono Hendarto, Retno Asti Werdhani, Meita Dhamayanti, Retno Pudjiati, Lathifah Hanum, Affan Naufal, Susan M. Sawyer

**Affiliations:** 1 Faculty of Medicine, Universitas Pelita Harapan, Bencongan, Kelapa Dua, Tangerang, Banten, Indonesia; 2 Faculty of Medicine Universitas Indonesia, Department of Child Health, Jakarta, Indonesia; 3 Faculty of Medicine Universitas Indonesia, Department of Community Medicine, Jakarta, Indonesia; 4 Faculty of Medicine Universitas Padjajaran, Department of Child Health, Pasteur, Bandung, West Java, Indonesia; 5 Faculty of Psychology Universitas Indonesia, Kampus UI, Depok, West Java, Indonesia; 6 Balaraja District Hospital, Tangerang, Banten, Indonesia; 7 Institute and Department of Paediatrics, Centre for Adolescent Health, Royal Children’s Hospital, Murdoch Children’s Research, University of Melbourne, Parkville VIC, Australia; University of Sharjah, UNITED ARAB EMIRATES

## Abstract

**Background:**

Obesity is a growing public health and clinical concern, worldwide. In many countries, including Indonesia, health professionals lack the capacity to promote behavior change around obesity prevention and management, especially with adolescents for whom a wider set of communication skills are required. This study describes the theoretical basis, approach to development of content, use and satisfaction of an internet-based educational intervention designed to improve the quality of health professional weight management counseling with adolescents in Indonesia.

**Methods:**

This study is part of an exploratory sequential mixed methods design which was undertaken from 2020 to 2022. Following a needs analysis, an internet-based training resource was developed, informed by constructive alignment theory and active learning principles. Using both synchronous and asynchronous approaches over a four-week pilot study, a weekly interactive session was held online, using multifaceted training materials housed on a website (https://ramahremaja.id). The training resource was then tested in a two-arm study involving health professionals from 17 of 34 provinces across Indonesia.

**Results:**

Sixty-four primary health professionals were recruited for the two-arm study. The completion rate for reviewing all materials and assignments on the website was 72% and the online meeting participation rate was 78%. Participants were highly positive about the clarity of the training material and the appropriateness of the delivery methods. The main challenges related to poor internet literacy and interrupted internet connectivity.

**Conclusion:**

Designed to support weight management in adolescents, this internet-based training program shows potential for enhancing Indonesian health professional behavior-change counseling skills.

## Introduction

Obesity is increasingly appreciated as a major global health problem [1]. Despite greater awareness over the past 25 years, its prevalence is steeply increasing in developing countries, especially in children and adolescents [2]. In Indonesia, a 2018 national survey showed that there were 19.8% or almost ten million adolescents with overweight or obesity [3], with expectations that this rate will have increased further due to COVID-19 pandemic restrictions [4]. Adolescent obesity is a major risk factor for later cardiovascular-metabolic disease, a group of disorders that already have the highest burden of any disease in Indonesia [5,6]. Yet in terms of obesity prevention, adolescence is also a key period of biological, social, and behavior change which lays the foundation for healthy lifestyles [7,8].

Given the enormous clinical burden posed by the prevalence of obesity, innovative prevention and care-delivery strategies are urgently needed [9]. Behavior modification approaches such as Motivational Interviewing (MI) are the most frequently recommended interventions for health professionals to use in response to patients with established weight concerns [10]. However, counseling interventions to address behavior-related health problems such as overweight and obesity are underutilized in healthcare settings. This is especially in low- and middle-income countries where the need to build capacity to address overweight and obesity is compounded by a poorly qualified health workforce [11–13].

In Indonesia, a qualitative study revealed that counseling quality was a prominent weakness of adolescent health services [12]. Heightening this challenge, clinical efforts to address overweight and obesity in adolescents need to be embedded within knowledge of adolescent growth and development, adolescents’ needs and capabilities, and the skills to practice effectively with both adolescents and their parents [14,15]. These efforts also require reasonable nutrition knowledge, yet a recent systematic review showed that nutrition education is poorly incorporated into medical education, regardless of country, setting or developmental focus [16].

We set out to develop a training program to address this gap in Indonesia, a country with a population of more than 275 million people spread over an archipelago of 17,508 islands [17]. In the context of the COVID-19 pandemic, we developed an internet-based training program that used both synchronous and asynchronous methods (blended learning) to improve the basic knowledge and counselling skills around behavior change in adolescents with overweight and obesity. Here we describe the theoretical basis of the training resource as well as its structure, content and format. We also describe the findings from the pilot program of how participants used the material and their satisfaction with it.

## Methods

The intervention was developed using an exploratory sequential mixed method design. A needs assessment study in adolescents and parents from three provinces in Indonesia has been previously published [18]. That study informed the content of the online training which was developed to improve health professionals’ basic knowledge and counseling skills around behavior change in adolescent patients using both synchronous and asynchronous methods. The current study was conducted according to the Declaration of Helsinki and conformed with the ethical standards of the Ethics Committee of Fakultas Kedokteran Universitas Indonesia (approval number 829a/UN2.F1/ETIK/PPM.00.02/2021)

### Theoretical basis of the training program

Walsh and McPhee’s System Model for clinical preventive care describes two major issues in providing behavioral change counseling for weight management in primary care settings from the health professional’s perspective. The first is the capacity of health professionals to provide services and the second is the workload of health professionals in primary care [19]. Capacity is influenced by the knowledge, skills and self-efficacy of health professionals [20].

In Indonesia, the current capacity of health professionals to address weight management is expected to be low: globally, there is little focus on this topic in health education in low- and middle-income countries like Indonesia, and little evidence that the principles of adolescent health and nutrition education have been widely incorporated into medical and nursing education [16]. Self-efficacy to counsel patients around healthy habits is also influenced by health professionals’ own health status and behaviors. Health workers with favorable personal health behaviors are more likely to counsel patients about healthy lifestyles than those with less favorable lifestyles and perceived low self-efficacy in nutrition care [16,19]. Consistent with the Walsh and McPhee framing, there are also competing demands in primary care settings in Indonesia which influence the time that can be spent with patients [21].

**Fig 1:**
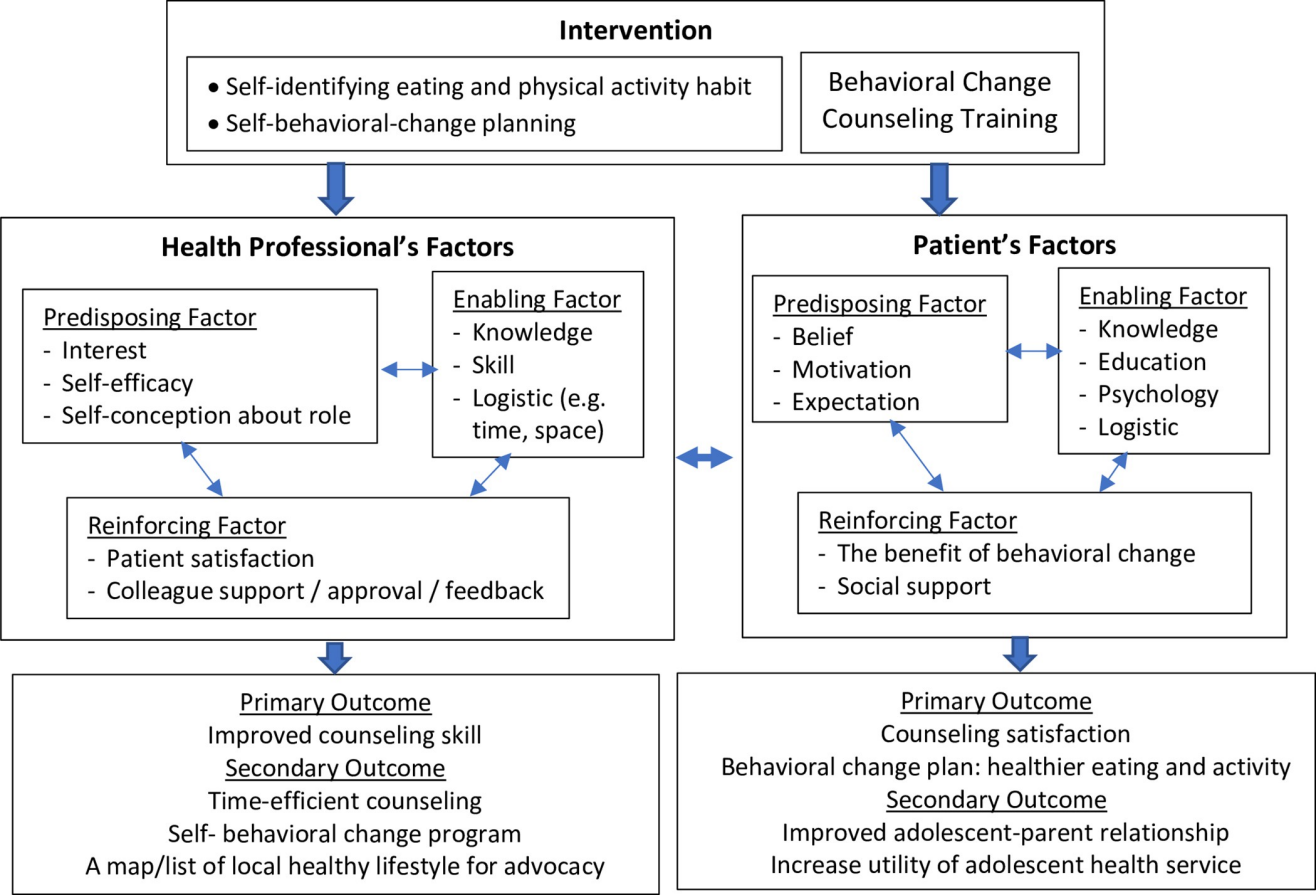
The intervention model based on Walsh and McPhee system model [19].

The following principles were identified to address these challenges. Firstly, the training would need to include comprehensive information on nutrition and physical activity, skill building to drive behavior change counseling using MI, and approaches to involving parents in the behavior change plan for their child. Secondly, the training would need to include activities for participants to identify their own eating and physical activity practices for them to develop a personal behavior change plan as a reflective tool to improve their self-efficacy in counseling patients around healthy lifestyle development. Thirdly, the time effectiveness that comes from having a framework and a set of counselling skills should be highlighted during the training. The conceptual model of the training was based on the Walsh and McPhee clinical preventive care model [19], as shown in the schema in Fig 1.

### Training program

Based on the needs assessment study involving adolescents and their parents from three provinces in Indonesia [18], the content of the training module and an accompanying guideline was developed by a team that consisted of a pediatrician with expertise in adolescent health, a public health nutritionist, a sports medicine specialist and a clinical psychologist with expertise in MI. The content was then reviewed by practitioners from adolescent health services (three nurses, three general practitioners), a group of relevant academics (two pediatricians, a sports medicine specialist, two child and adolescent clinical psychologists, and an epidemiologist), representatives from four Indonesian professional organizations (pediatric society, sports medicine society, family medicine society and clinical psychologist society), and representatives of the Ministry of Health Republic of Indonesia. Following feedback and revision, a website https://ramahremaja.id was then created to host the various documents which were sequenced as an interactive training course.

**Fig 2:**
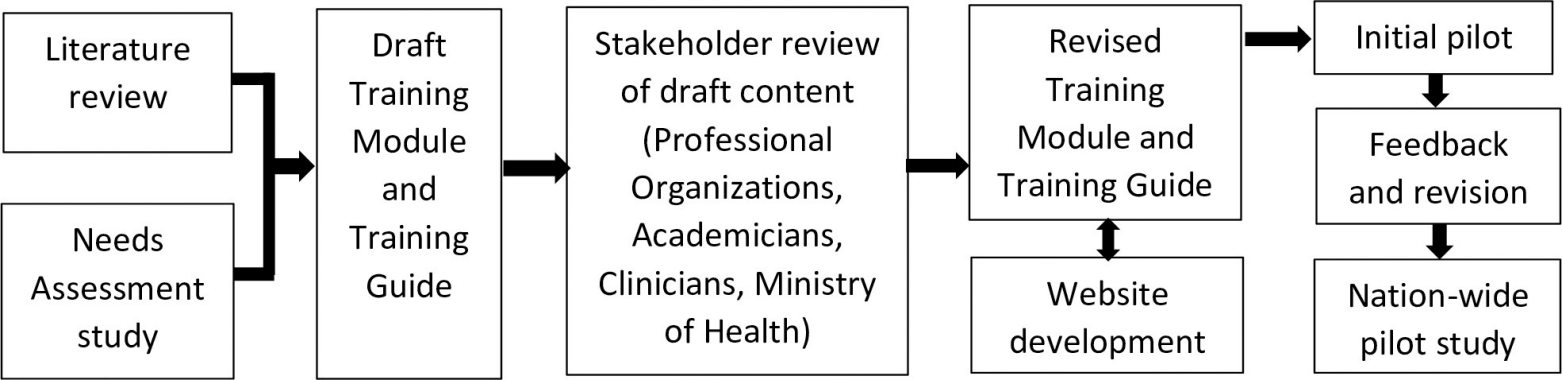
Training development sequence.

A pilot study was conducted with 11 health professionals (six nurses and five physicians appointed by the Ministry of Health Republic of Indonesia) to test the feasibility of the training and to identify areas needing modification. This initial pilot study resulted in various recommendations around both the content and method of delivery. The extent of negative feedback about the website from participants led to the decision to recruit a new online learning consultant and web developer. Following this initial pilot study, the training material was further refined. Subsequently, a two-arm pilot study was implemented to understand the training effectiveness (see Fig 2).

### Training content

Content development was based on constructive alignment theory in which there is intentional alignment of teaching design between the intended learning outcomes, teaching-learning activities and assessment tasks [22].

**Fig 3:**
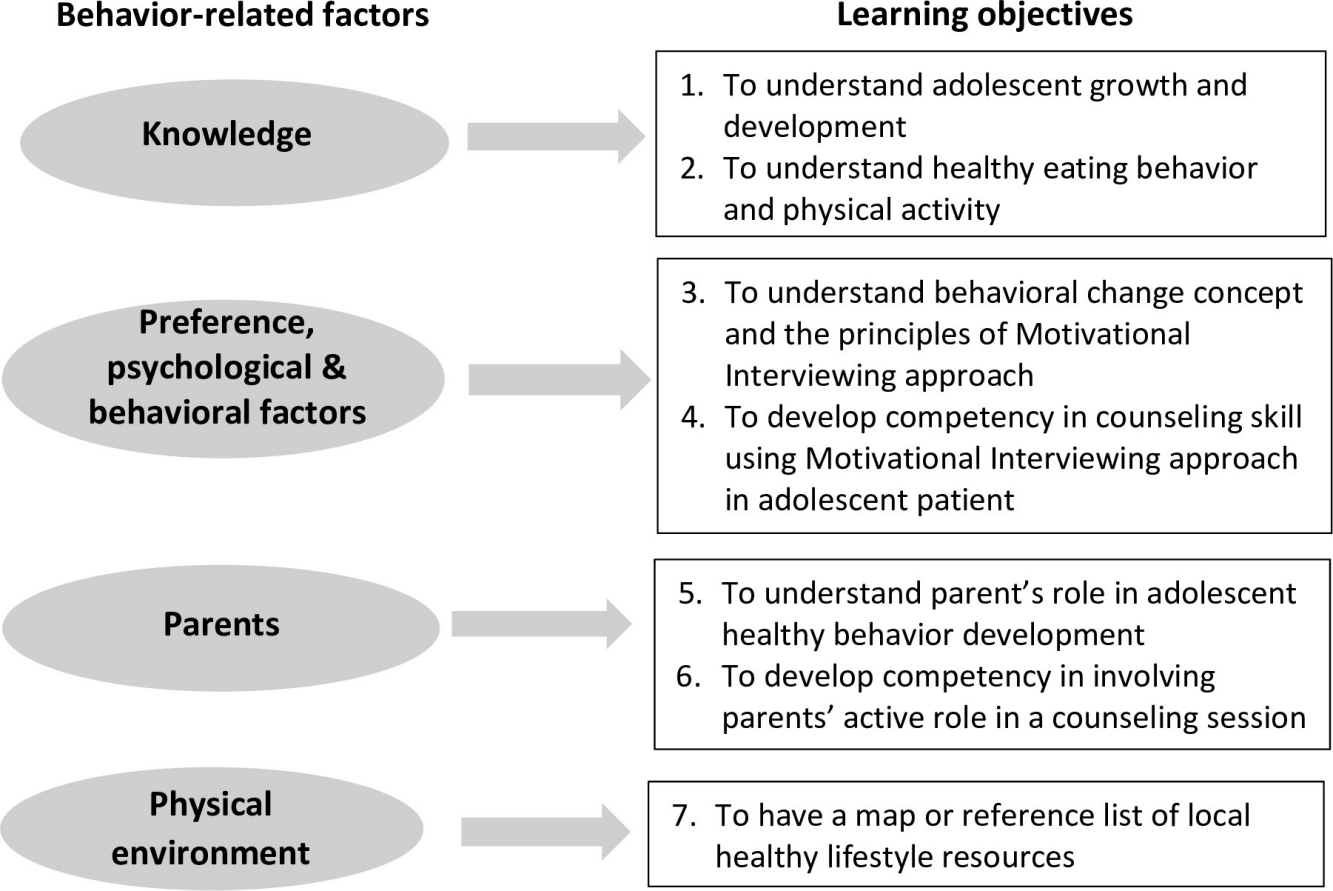
Map of learning objectives.

The development of the learning objectives was based around the framework that Neufeld and colleagues [23] recently developed to describe the modifiable aspects of overweight and obesity in adolescents. Our needs analysis affirmed that this framework is highly relevant for Indonesian adolescents and families, who had little interest in wanting to lead more healthy lifestyles and for whom non-supportive environments were major barriers for behavioral change [18]. MI was identified as an effective and efficient approach to address the individual factors around behavior change. MI is a patient-centered counseling technique that has the most evidence for the formation of healthy lifestyles, including for adolescent patients [10,24,25]. The learning objectives were for the health professional to address the modifiable factors which included individual factors (knowledge, preference, psychological and behavioral factors), the social environment (family / parents) and the physical environment (availability and accessibility of healthy lifestyle resources), as shown in Fig 3.

### Training activities

**Fig 4:**
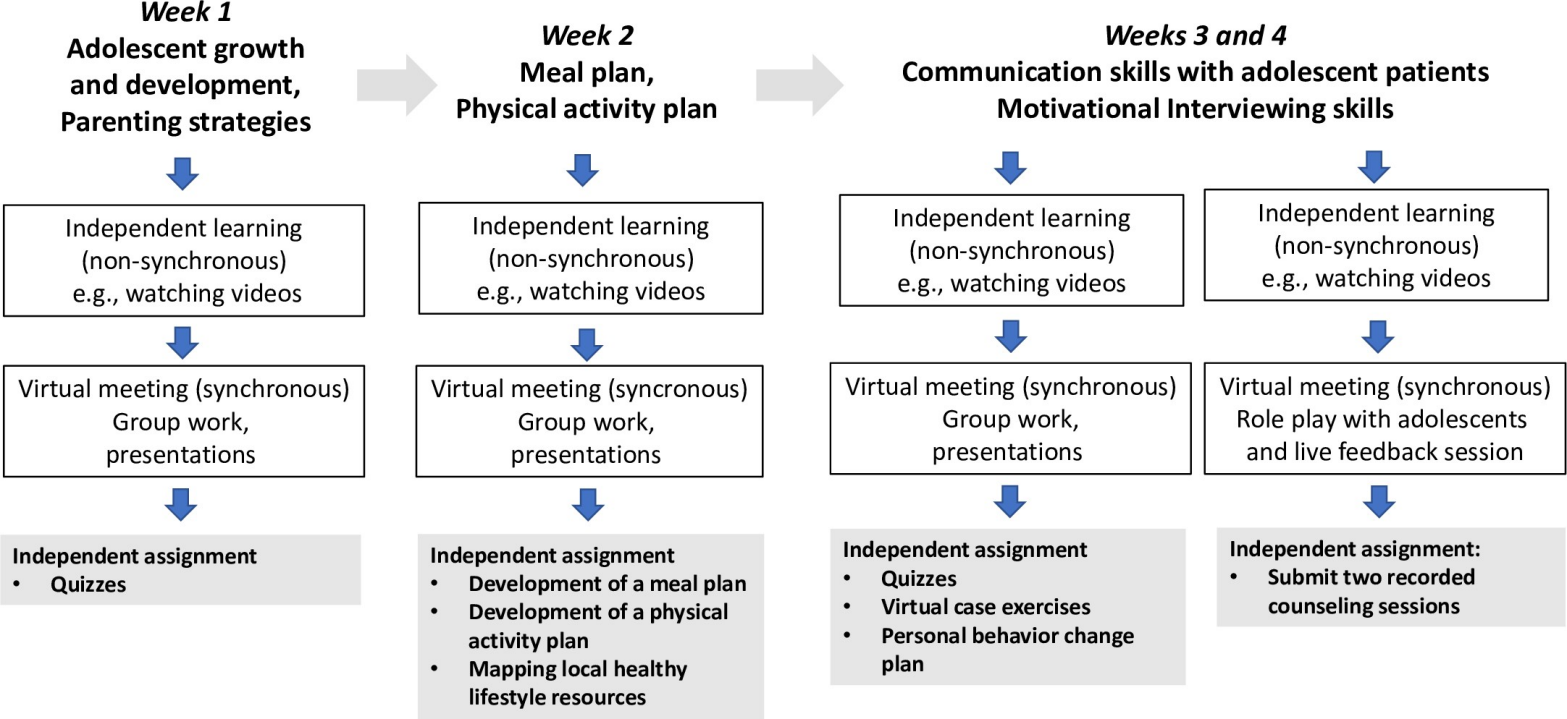
Training scheme flowchart.

The training activities were developed using the principals of active learning that require engagement in activities (writing, discussion, and presentation) and externalizing cognitive processes [26]. For independent learning (asynchronous) sessions, activities included watching videos, taking quizzes, the use of virtual cases as games, and written assignments. Training activities within the online meetings (synchronous sessions) included group work, group presentations and conducting counseling sessions with real adolescents. For each topic, participants were required to work through the available training material on the website before joining the online meeting. Rather than functioning as a lecturer, the facilitator’s role was intended to provide further explanations to guide participants’ understanding and give individual feedback on assignments and counseling sessions. The flow of the training program and its elements is outlined in Fig 4.

### Format

The program was hosted on a secure web server that was able to be accessed through an ordinary web browser (https://ramahremaja.id). The website used a learning management system that supported planning, implementation and access to all parts of the training for participants, facilitators and assessors. All participants created an account on the website which needed to be approved by the web administrator. After email verification, participants were welcomed with a written explanation, a video and an informed consent document for them to sign.

The online training navigation system was set to follow a certain sequence that required each participant to complete each session’s learning material, including assignments, before they were able to progress to the next part of the 4-session training program. The system also provided access to interactive features such as simulated virtual cases. Using their personal account, participants uploaded audio-taped counseling sessions to the website and could also use it to track their progress through the training.

A tracking system was also developed for facilitators to track the progress of each individual participant and the group of participants as a whole. The evaluation system was embedded within the website allowing facilitators and assessors to provide blinded individual quantitative and qualitative feedback that was directly accessible by individual participants.

### Two-arm pilot study

#### Study design and study participants

The subsequent pilot study consisted of a randomized trial of health care professionals who were randomized to either the intervention group or the control group based on their district. The Directorate of Nutrition, Child and Maternal Health, Ministry of Health was involved in the selection of provinces. With the expectation that participants had at least a basic level of understanding of adolescent health services, provinces were chosen on the basis that at least 50% of their primary health care centers provided specific health services for adolescents. Among 34 provinces, 18 provinces were identified, of which one declined to participate due to COVID 19-related workloads. From these 17 provinces, 64 health professionals from primary health care centers were nominated by provincial health offices to participate in the pilot study. Of these, 29 did not wish to participate and the district health officers replaced them, leading to a final sample of 29 physicians and 35 nurses.

After receiving the names of eligible participants, a researcher called each health professional to explain the study, which was followed by a formal letter from the principal investigator and the provincial health office. This included a written consent form and a baseline survey to test their knowledge of healthy eating, reported level of physical activity and behavior change counseling skills with adolescent patients. The survey took approximately 20 minutes to complete and included sociodemographic characteristics, and body weight and height. The Food Frequency Questionnaire (FFQ), one of the most widely used dietary assessment tools for capturing usual food intake [27], was administered to identify health professionals’ own food intake. The International Physical Activity Questionnaire (IPAQ) short version was used to measure physical activity recall, a globally used brief tool that is easy to administer. The Indonesian version has been validated and shows significant correlation with subjective scores of physical activity [28].

Each consenting participant was required to identify two adolescent patients with a BMI ≥ 85 percentile (WHO growth chart) with the goal of undertaking two counseling sessions with each of them, as well as with their parents. Each participant (health professional) provided a verbal explanation of the study to each adolescent and their parents, and subsequently obtained informed written consent (from parents) and assent (from adolescents) to audio-record the two counseling sessions. These counselling sessions could be undertaken in person or using telehealth facilities (zoom or video call), depending on the context of COVID-19 lockdowns and adolescent and parent preference.

Participants were then randomized to either the control or intervention group based on their district. The intervention group received the 4-week training immediately after their first counseling session with two adolescents and their parents. In contrast, the control group received the 4-week training after conducting the second counseling session.

#### Evaluation of training

An evaluation form was completed by participants around that week’s training materials and the online interactive session. Each aspect of the training material was evaluated quantitatively and qualitatively for clarity of content and attractiveness of delivery method. Participants were encouraged to be critical with their review. Response options used a 5-point Likert scale, with 5 being the most positive response. Summary quotes are also presented, identified by discipline (physician or nurse) and participant number. Knowledge and counseling skills of the participants (health professionals) were assessed with the goal of understanding the effectiveness of training. The knowledge and the counseling skill assessment were done using validated assessment tools designed specifically, based on the learning objective of the training. The counseling quality assessment was performed by trained professional raters (clinical psychologists) whose individual feedback was provided to participants through the website. The evaluation of change in participants’ knowledge and counselling skills is to be reported in a subsequent manuscript.

## Results

### Characteristics of participating health professionals

Sixty-four health professionals (39 physicians, 35 nurses) participated in the substantive pilot study of the training program. Most participants were female (52 participants). The largest age-group of participants was 25–34 years old (n = 33), followed by 35–44 years (n = 23). There were only 2 participants aged 20–24 years old and 6 participants above 45 years old. Fourteen participants had a history of prior training related to adolescent health services.

### Training participation rate

The mean completion rate for reviewing all materials and assignments on the website from the 64 participants was 72% (range 22–100%). Over half (n = 34, 53%) of the health professionals completed more than 70% of the website tasks, while around one in ten (n = 7, 11%) completed less than 30% of the tasks. Participation in the online meetings was 78% (range 72–85%).

### Feedback from participants

High scores were obtained for both clarity of content (4.4 out of 5) and attractiveness of delivery method (4.4 out of 5). The explanatory videos and counseling simulation videos were the most highly rated training materials, while written modules were the least preferred form of training material. At the end of the training, general satisfaction and willingness to participate in similar training were also rated highly, with scores of 4.5 and 4.6, respectively (see Table 1).

**Table 1:** Assessment scores in 5-point Likert scale.

	Training Material	Method of Delivery
**Topic 1 (week 1)**		4.27
• Adolescent growth and development	4.29	
• Parenting of adolescents	4.29	
**Topic 2 (week 2)**		4.40
• Healthy eating	4.35	
• Physical activity	4.52	
**Topic 3 (week 3)**		4.23
• Counseling technique 1 (Basic Principles of Motivational Interviewing)	4.5	
**Topic 4 (week 4)**		4.6
• Counseling technique 2 (Motivational Interviewing Role Play	4.6	
**Overall assessment of 4-week program**	4.5	
**Willingness to participate in other training at ramahremaja.id**	4.6	

Most participants also provided written comments, which were overwhelmingly positive:

*“The topic was interesting*, *there were games*, *there was a hands-on practice of making menus and a physical activities schedule and there were direct roleplays with adolescents and live feedback from facilitators”* (Nurse, participant 7)

*“The training was very well organized and neat*, *so the participants were ready for the zoom session with two-way activities”* (Physician, participant 10)

*“Everything that was presented in the training*, *starting from the website*, *facilitators*, *modules*, *etc*. *was very well prepared and clear even though it’s only virtual*” (Physician, participant 22)

These comments reinforce the enjoyment of the active learning approaches, and the appreciation by participants of the individual feedback from clinical psychologists.

Two main obstacles were apparent during the training: poor internet literacy and internet connection. The mechanism for uploading counseling recordings, and accessing and uploading assignments to the website was challenging for some participants. Despite individual clarification by phone (in addition to the video explanations and detailed instructions within the website), four participants emailed their audio-tape counseling sessions to the facilitator instead of uploading them to the website system. Poor internet connection was experienced by some participants, especially those from Sulawesi and Nusa Tenggara.

*“I couldn’t open the videos on the website*, *maybe because of the high resolution”* (Physician, West Nusa Tenggara participants)

*“I was constrained by the internet connection as it crashed often”* (Nurse, West Sulawesi Participants)

The video resolution was subsequently reduced to facilitate participation. Internet connection was also an obstacle during the online meetings, with some participants only able to participate intermittently because of online signal instability, especially during rainy weather. This limited the extent that some participants were able to actively engage in the discussion.

## Discussion

Based on our knowledge, this is the first internet-based training for the health workforce in Indonesia that aims to enhance communication skills with adolescent patients around behavior change. The training intervention is a theory-driven model for health professional education that was applied using an interactive multi-media format that was designed to enhance health professionals’ communication with adolescent patients regarding weight management. The training was liked by most participants on the basis of its interactive format and flexibility in the learning schedule, consistent with reports from adolescent weight management training programs in high income countries [29,30].

This training was designed using both asynchronous and synchronous methods to encourage participant engagement and completion of training materials. A similar training program around the pilot implementation of the ICATT (IMCI—Integrated Management of Childhood Illnesses—Computerized Adaptation and Training Tool) was carried out by health workers independently in several developing countries, including Indonesia. It was not considered particularly successful because it required strong internal motivation [31,32]. The feedback we obtained from participants in this training program suggested that the weekly interactive meeting that contained group discussion activities and presentations stimulated participants to watch and to learn the material on the website beforehand. Participants also shared that the active interaction with the other participants from various provinces as well as the facilitators also helped to engage their interest and promoted their motivation to learn. These findings are consistent with meta-analyses by Wandera et al [33] and Liu et al [34] who identified that blended learning approaches where training is accompanied by meetings both inside and outside one’s network are more effective when compared to self-directed online training. These meta-analyses also suggested that online meetings and live discussions increase the number of hours participants spend learning, help clarify the task or learning instructions and enrich understandings [33,34].

A major strength of this training program was that it was carefully developed using the constructive alignment method and active learning principle which enhanced the clarity and interest around the learning objectives and learning activities. As with other internet-based interventions in resource poor countries, poor internet connectivity and low internet literacy of participants was a limitation [35]. All of the videos were subsequently downgraded to the lowest resolution and greater visual explanations were provided with an expectation that not all participants were able to view the video materials. However this pilot study has shown that conducting an internet-based training for health workers in a large archipelago like Indonesia is possible. Due to the instability of internet connection in particularly remote areas, a hybrid learning model where participants are invited to travel to a place with better internet connectivity may help overcome this issue. One important learning gained from the development of this training program was that, as appreciated by others [36], establishing a web-based application for online learning is a complex task and requires experienced web developers. The extent of feedback from our initial pilot study confirmed that we needed to change the web developer and rebuild the website, which was a major challenge that could have been avoided if we had appreciated how important it was for the web developer to have specific expertise in online learning systems at the outset.

### Conclusion and implications

Given the importance of primary care as a setting for preventive care for adolescents, developing acceptable, scalable training interventions for health professionals to address weight management through counselling is important. The development of this intervention provides low-cost training that covers specific issues around weight management counseling of adolescent patients. The distinctive elements of this training program were the inclusion of parenting strategies to address the social environment and activities to map healthy lifestyle resources to address the physical environment. Adding to this, participants were engaged in activities that promoted reflection around their personal behaviors with the goal of improving their self-efficacy for behavior change counseling. The hybrid method and active learning approaches throughout the training which included role plays with real adolescents, together with individual feedback, are thought to have enhanced participant engagement, and which resulted in a high level of acceptability by health professionals. The next stage in the evaluation of this training program is to assess the extent to which it improves health professional knowledge and skills, and in due course, what impact this might have on adolescents who are over their healthiest weight.
